# Age Limits for Institutional Posts

**Published:** 1914-04-04

**Authors:** 


### The Hospital

The Workers' Newspaper of Administrative Medicine and Institutional Life, Administration, National Insurance and Health.

No. 1419, Vol. LVI. SATURDAY, APRIL 4, 1914. PRICE ONE PENNY.

### AGE LIMITS FOR INSTITUTIONAL POSTS.

Some considerable degree of feeling has been aroused in Glasgow, we learn, by the terms of an advertisement which has appeared inviting applications for two vacant posts at the Royal Hospital for Sick Children in that city. In each case the stipulation is made that candidates must not be over forty years of age, and this has aroused a certain amount of indignation amongst, at any rate, some who have already passed that milestone of maturity and possibly amongst others who have yet to reach it.

The general principle involved in the "too old at forty" issue is apt to be confused by personal consideration and personalities in concrete examples of its application; and, indeed, apart from such really irrelevant matters, there is a good deal to be said on both sides of the question. It is easy to state a strong case in support of age limits of this kind: it is just about as easy to quote scores of cases in which they would operate unjustly to the candidates, or disadvantageously to the institution. As far as the visiting medical staff are concerned, a rule of this kind is in operation in some few of the largest teaching hospitals, though in the majority, probably, it is not. Even where no such rule is in force, in practice it is very rare for a man to obtain his first appointment on the visiting medical staff when once he has passed two score years, and most of the men thus appointed are under thirty-five. Still, it would not be difficult to give the names of three or four well-known consultants who had passed forty before they got their foot on the institutional ladder; one, if not two, of the most recent appointments at a leading London general hospital, with a very large medical school, could be quoted in point. In special hospitals and in county and provincial hospitals this does not hold good; and it would be a great mistake to apply the rule to staff appointments at such institutions. No one, of course, would think of adopting it for appointments to the in-patient staff at any hospital: it is only for the assistant surgeonships and physicianships that it can be pronounced useful.

Turning to the other departments of institutional life—and it was, we gather, not a medical vacancy which was being advertised in Glasgow—much depends on the character of the work which is being demanded. No hospital, except possibly a lying-in hospital, would accept probationer nurses over the age of forty years; nor is it desirable that training should begin in excess of that age. Clerkships in the secretarial department, again, would naturally be unsuitable for any but young men to take up; the necessary years of training demand an adaptability which is peculiar to youth and is lost in later life. House surgeons and house physicians are almost always quite young men, and it is desirable, as a rule, that they should be: the only experiment with a man of over forty which has come directly under our notice in recent years ended in a great deal of friction and loss of efficient working. For higher administrative posts, such as those of secretary, medical superintendent, matron, housekeeper, or steward, an age limit, if imposed at all, should not be made as low as forty; forty-five or even fifty would be a more reasonable limit.

With regard to appointments such as engineer, carpenter, porter, *post-mortem* attendant, male nurse, liftman, and similar offices, it is not so easy to lay down hard-and-fast rules. Many of these appointments require long experience and special training, and it is, therefore, desirable to get a young man, so that he may be both teachable and likely to give many years' service when taught. This applies particularly to operating-theatre porters, casualty-room porters, and male nurses; yet many most valuable institutional servants in these capacities have not been "caught young," but have taken up the work in middle age. Much depends on a man's previous career: a few years in the Army or Navy is the best of all possible guarantees that a man will readily pick up what is required of him for such posts. When this has been admitted, it is, nevertheless, true that institutional servants should be chosen above all things with a view to permanency if possible; and a young man naturally has more years of useful service to give than one over forty.

B

While sympathising with those men between forty and fifty who by dint of temperate life and clean living feel themselves equal in physical energy, and superior in experience and tact, to their younger rivals, we do not feel inclined to blame institutional managers who advertise for men of under forty. The rule must operate harshly in individual cases, and may exclude men who would do most valuable service; but it is impossible to condemn it out of hand as impolitic, for in the long run it probably protects the best interests of the institution. Neither, however, do we blame those institutional managers who do not adopt this rule, provided they are duly impressed with the danger of saddling their institution with a servant whose age and previous career may render him of doubtful utility. If anyone wants proof of the splendid and prolonged service which middle-aged, and even old, men of the right stamp can render to a hospital, we would commend to their notice the details which were given in these columns not long ago (The Hospital, January 31, p. 467) of three retiring institutional workers whose records would have been remarkable, even if they had started as young men, but were quite phenomenal considering that all three were middle-aged before they were appointed. It will be seen that men are not necessarily too old at forty to begin institutional careers, but that it is only in exceptional cases that it is advisable to appoint them after that age.

